# Robot-Assisted Laparoscopic Seminal Vesicle Cystadenoma Excision

**DOI:** 10.1089/cren.2015.0011

**Published:** 2015-12-01

**Authors:** Omer Burak Argun, Panagiotis Mourmouris, İlter Tufek, Yesim Saglican, Can Obek, Ali Riza Kural

**Affiliations:** ^1^ Department of Urology, Maslak Acibadem Hospital, Acibadem University, Istanbul, Turkey.; ^2^ Department of Pathology, Maslak Acibadem Hospital, Acibadem University, Istanbul, Turkey.; ^3^ Department of Urology, Cerrahpasa School of Medicine, İstanbul University, Istanbul, Turkey.

## Abstract

***Background:*** Cystadenoma is an extremely rare benign tumor of the seminal vesicle. Diagnosis of these tumors and differential diagnosis from malignant ones may be challenging since most of the time symptoms do not occur. Management of these tumors remains debatable due to the limited data in the literature. We present the first robot-assisted laparoscopic excision of a cystadenoma of the seminal vesicle.

***Case Presentation:*** A 48-year-old man presented with diminished ejaculate volume and a 3.5 cm right seminal vesicle mass, which increased its size at 6 cm after the 3-month period. Transrectal ultrasound-guided biopsy revealed no malignancy. Robot-assisted laparoscopic excision of the tumor was performed. Port placement was the same as robot-assisted radical prostatectomy. Operative time and estimated blood loss were 240 minutes and 200 mL, respectively. Patient was discharged on postoperative day 3 without any complications. Final histopathologic examination revealed cystadenoma of the seminal vesicle.

***Conclusion:*** Surgical intervention may be considered when a cystadenoma of the seminal vesicle is diagnosed and symptoms or tumor growth occurs. Robot-assisted laparoscopic excision is an alternative in the management of these rare tumors.

## Introduction and Background

Primary or secondary tumors of seminal vesicle are rare conditions and they can be either benign or malignant lesions, with the last to be extremely difficult to diagnose due to the lack of symptoms. Cystadenoma is an extremely rare benign tumor of the seminal vesicle with only 15 cases reported so far in the English literature. The management for these tumors remains debatable since the reports in the literature are limited; nevertheless, surgical intervention is usually needed when symptoms or increasing size occurs. Various approaches have been proposed for the treatment of these tumors until today. We present the first robot-assisted laparoscopic excision of a cystadenoma of the seminal vesicle.

## Case Presentation

### Clinical history

**FIG. 1.**
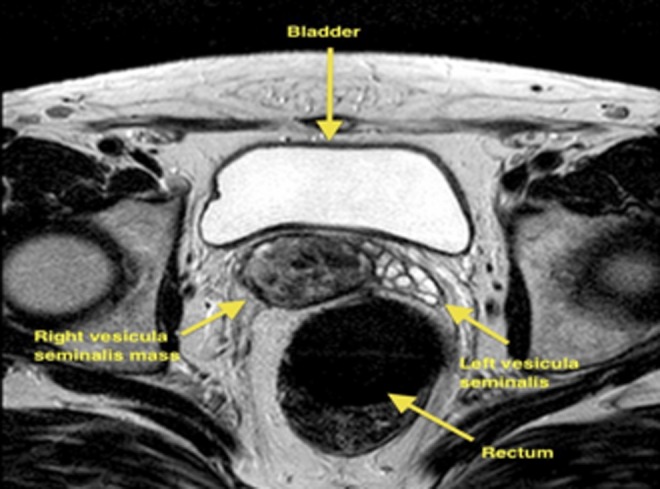
MRI demonstrating a right seminal vesicular mass between the bladder and rectum.

A 48-year-old man presented with diminished ejaculate volume lasting for several years. The patient underwent transrectal ultrasonography (TRUS), which identified a 3.5 cm solid mass with cystic and solid areas arising from the right seminal vesicle (Fig. 1). These tumors can cause hematuria, obstructive urinary symptoms, painful ejaculation, and diminished ejaculate volume or they can be asymptomatic.^1^ In our case, the only complaint of the patient was decreased ejaculate volume.

### Physical examination

The patient underwent a digital rectal examination with no specific findings. MRI was then planned, which revealed a 3.5 cm complex cystic mass arising from the right seminal vesicle. To exclude malignancy, a TRUS biopsy of the tumor was planned, but due to the patient's refusal, close follow-up was scheduled.

### Diagnosis

After 3 months, a new MRI revealed a growth of the tumor from 3.5 cm to 6 cm. Although benign tumors of the seminal vesicles do occur more frequently, the mass has increased its size in a short follow-up period, and therefore, malignancy had to be excluded to plan the therapeutic strategy for the patient. Primary malignant tumors are extremely difficult to diagnose,^2^ but they may require more radical procedures. Due to the above-mentioned reasons, a TRUS biopsy of the mass was arranged with a pathology report of the specimen revealing no malignancy but being inconclusive of the type of the tumor.

### Intervention

**FIG. 2.**
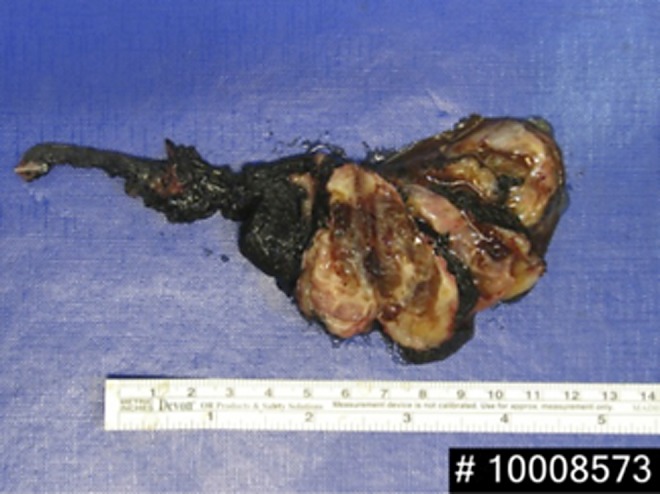
Surgical specimen: multilobulated cystic mass.

Management of benign tumors of the seminal vesicle remains debatable due to the limited data in the literature. Nevertheless, the presence of symptoms, which often relates to the tumor size, seems to be the trigger point for surgical intervention in most cases reported. The robot-assisted laparoscopic approach was chosen for the surgical excision of the tumor with the use of a four-arm Da Vinci Si system. Preoperatively, a 4.8F Double-J catheter was placed into the right ureter for easier ureteral identification. Port configuration was similar to transperitoneal robot-assisted radical prostatectomy. The peritoneum was incised over the seminal vesicle, as in the Montsouris technique. Right vas deferens was identified, dissected, and divided. Weck clips were used to control the vessels avoiding thermal damage to the ureter. There were severe adhesions blocking the posterior side of the seminal vesicle. The right seminal vesicle was dissected out carefully with the athermal technique. Then, the anterior aspect of the seminal vesicle was exposed. Once the specimen was freed, it was taken out through a 12 mm assistant port within a 10 mm organ retrieval bag (Fig. 2). A frozen section of the specimen revealed no malignancy. A Hasson balloon trocar was inserted to reestablish pneumoperitoneum. The cavity was filled with saline to perform the bubble test, ruling out any rectum injury. The peritoneum was closed, and a Jackson-Pratt drain was placed. Operative time and estimated blood loss were 240 minutes and 200 mL, respectively.

### Follow-up

**FIG. 3.**
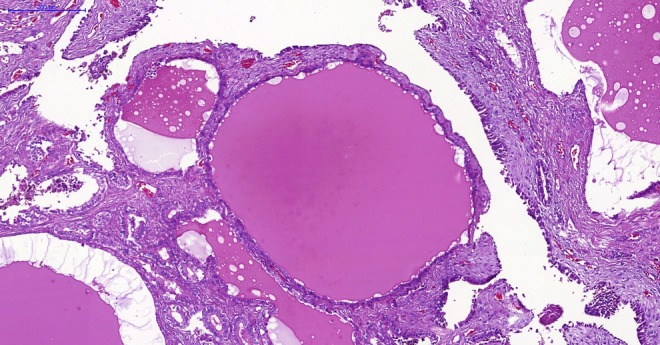
Histologic view of this cystadenoma of the seminal vesicle (hematoxylin and eosin staining). Multiple cysts of varying sizes and shapes are filled with homogeneous eosinophilic material, with no malignant features. Scale bar = 200 μm.

The postoperative period was uneventful, and the patient was discharged on postoperative day 3. The final pathology report revealed a benign cystadenoma of the right seminal vesicle (Fig. 3).

### Outcomes

The patient was followed for a 1-year period. During this period, he had normal erections, effective sexual intercourse, and complained for no other symptom except ejaculate volume, which remained diminished in the follow-up period.

## Discussion and Literature Review

Due to the rarity of these tumors, no standard surgical approach can be concluded. Various open surgical techniques have been described in the literature, including transperineal, transvesical, paravesical, retrovesical, and transcoccygeal approaches.^3^ There are limited and sparse data for the use of minimal invasive techniques in seminal vesicle cystadenoma excision. The only available data so far in the literature come from Zhu and colleagues, who performed a laparoscopic excision of an 8.8 cm left seminal vesicle cystadenoma.^4^ They reported 125 minutes surgery time and 120 mL estimated blood loss. The patient was free of symptoms for a 10-month follow-up period. To our knowledge, no robot-assisted laparoscopic excision of cystadenoma of the seminal vesicle has been reported so far. Robotic assistance eases resection with the advantage of articulated robotic arms and 7 degrees of freedom of robotic instruments. This aids in precise dissection and in avoiding any injury to adjacent organs. Both 0° and 30° lenses can be used during the procedure. In our case, a 30° lens was used in the upward position during the posterior dissection that improved view and offered optimal exposure of the tumor and the seminal vesicle. An intraoperative biopsy may be mandatory after tumor excision, especially if the preoperative biopsy was inconclusive, since a possible malignant pathology in the frozen section specimen can lead to a more radical procedure. In conclusion, cystadenoma of the seminal vesicle is a rare entity and a surgical intervention may be considered in the case of symptoms or tumor growth. Robot-assisted laparoscopic excision can be an alternative in the surgical management of these rare tumors.

## Abbreviations Used

MRI: magnetic resonance imaging
TRUS: transrectal ultrasonography
